# Protocol for antibody-based m6A sequencing of human postmortem brain tissues

**DOI:** 10.1016/j.xpro.2025.104149

**Published:** 2025-10-17

**Authors:** Haruka Mitsuhashi, Naguib Mechawar, Corina Nagy, Gustavo Turecki

**Affiliations:** 1 McGill Group for Suicide Studies, Douglas Mental Health University Institute, McGill University, Montreal, QC H4H 1R3, Canada; 2 Integrated Program in Neuroscience, McGill University, Montreal, QC H3A 0G4, Canada; 3 Department of Psychiatry, McGill University, Montreal, QC H3A 0G4, Canada

**Keywords:** Genomics, Antibody, Neuroscience

## Abstract

Here, we present an optimized N6-methyladenosine (m6A) immunoprecipitation sequencing for human postmortem brain tissue, building on previously established approaches. We describe steps for RNA extraction, RNA shearing, RNA immunoprecipitation, and library preparation. The protocol includes optimization data for each step, such as m6A profiles from total RNA versus poly(A) RNA as an input, a comparison of different commercial m6A antibodies, and immunoprecipitation with varying RNA/antibody ratios. This protocol allows reliable capture of m6A peaks from human postmortem brain tissue.

For complete details on the use and execution of this protocol, please refer to Mitsuhashi et al.^1^

## Highlights

• Steps for RNA extraction from human postmortem brain tissues
• Instructions for mechanical RNA shearing prior to RNA immunoprecipitation
• Procedures on RNA-IP with an m6A antibody followed by library preparation

## Abstract (editor highlights)

Publisher’s note: Undertaking any experimental protocol requires adherence to local institutional guidelines for laboratory safety and ethics.

## Graphical abstract

**Figure:**
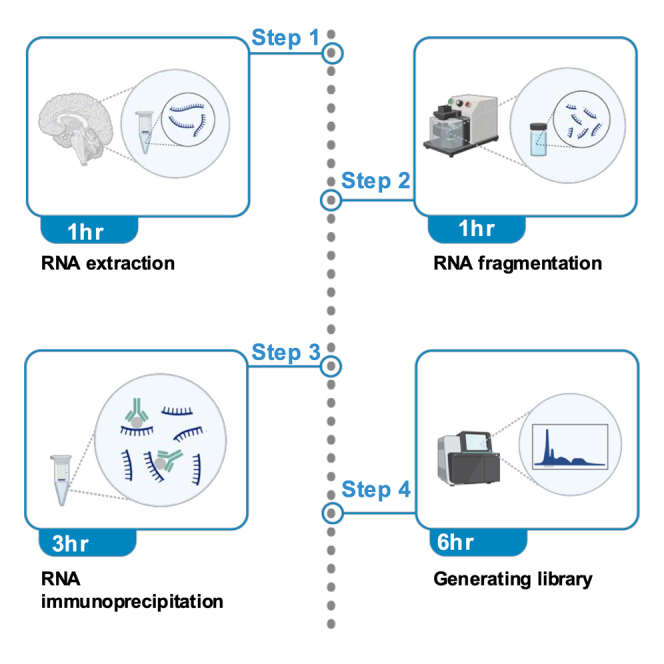

## Before you begin

The most widely used method for mapping m6A across the transcriptome is antibody-based enrichment, known as m6A sequencing (m6A-seq) or MeRIP-seq.^2^^,^^3^ This approach involves the immunoprecipitation of m6A fragments using an m6A-specific antibody, followed by sequencing, which allows for the identification of m6A peaks at a resolution of 100–200 nucleotides. However, the application of the original method was limited by the large amount of total RNA required as input. To address this limitation, several low-input methods have been developed, enabling the application to limited tissues, such as human postmortem brain samples.^4^^,^^5^ Another limitation of the antibody-based method is that the capture of m6A peaks largely depends on the affinity of the antibody used.^4^^,^^6^ Previous studies comparing m6A-seq profiles generated using different m6A antibodies found a limited overlap of m6A peaks between three antibodies with just 60% and differences in m6A motif enrichment.^4^ The bioinformatic tools used to analyze m6A-seq data, including peak calling, represent another means of potential variable across results.^6^ Indeed, several peak calling methods have been developed for m6A-seq data to account for the sources of variability associated with using transcript abundance as a background.^7^^,^^8^

**Figure 1:**
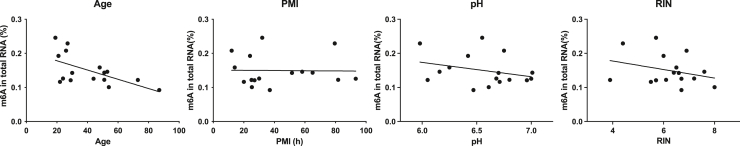
Global m6A levels in post-mortem brain tissues Global m6A levels were measured by m6A RNA Methylation Assay Kit (Abcam: ab185912) according to manufacture instruction. Correlation analyses showed that global m6A levels were not significantly affected by PMI, pH, or RIN. However, there was a trend of decreasing global m6A levels with age.

Human postmortem brain tissue is a valuable resource allowing us to directly study the molecular underpinning of the human brain. However, studies leveraging postmortem tissue face an important challenge of autolysis, often associated with longer postmortem intervals (PMI). Several studies have carefully examined the influence of PMI and tissue pH, and their impact on RNA, miRNA, protein, and epigenetic integrity.^9^^,^^10^ Messenger RNA is particularly prone to degradation due to its single-strand structure, and therefore, preserving the RNA quality is critical for studying human postmortem brain tissue.^11^ To assess the postmortem stability of m6A, we quantified global m6A levels in postmortem brain tissues (n=16) with varying PMI, RNA integrity numbers (RIN), and pH levels. No significant correlations were observed between the global m6A levels and these measures of molecular integrity. The global m6A levels remained relatively consistent across different PMI (Rˆ2=0.0003), RIN (Rˆ2=0.0853), and pH (Rˆ2=0.0896), suggesting that m6A is relatively stable in the postmortem brain (Figure 1). Overall, we showed feasibility of applying m6A-seq on human postmortem brain tissues.

### Innovation

This m6A-seq protocol is specifically optimized for human postmortem brain tissues following previously developed low-input protocol.^4^ During the process of optimization, we compared two publicly available m6A-seq protocols that differ primarily in their use of total RNA or poly-A RNA as input. We also established RNA fragmentation parameters using mechanical shearing. Additionally, we examined the characteristics of m6A peaks generated using different commercially available m6A antibodies. We further optimized immunoprecipitation conditions, including RNA and antibody ratio, using m6A antibody from Synaptic Systems to achieve higher specificity. This protocol allows the reliable capture of m6A peaks from human postmortem brain tissue.

### Institutional permissions

All experiments involving the use of human samples must be performed in accordance with the relevant institutional and national regulations. Use of postmortem tissues was approved by the Institutional Review Board of the Douglas Hospital.

## Key resources table

**Table:** 

REAGENT or RESOURCE	SOURCE	IDENTIFIER
**Antibodies**		

m6A antibody (1 mg/mL)	Synaptic Systems	Cat#202003

**Biological samples**		

Frozen postmortem human brain tissue	Douglas-Bell Canada Brain Bank	NA

**Chemicals, peptides, and recombinant proteins**		

Protein-A magnetic beads	Thermo Fisher Scientific	Cat#10001D
Protein-G magnetic beads	Thermo Fisher Scientific	Cat#10003D
RNasin Plus RNase inhibitor	Promega	Cat#2611
Buffer RLT	QIAGEN	Cat#79216
Igepal CA-630	Sigma-Aldrich	Cat#18896
Tris-HCl (pH 7.5, 1 M)	Sigma-Aldrich	Cat#T2663
NaCl (5 M)	Sigma-Aldrich	Cat#S6546
Ethanol 100%	Sigma-Aldrich	Cat#459836-500ML
Nuclease-free water	Sigma-Aldrich	Cat#3098

**Critical commercial assays**		

RNeasy lipid tissue mini kit	QIAGEN	Cat#74804
RNA Clean and Concentrator	Zymo Research	Cat#R1015
SMARTer stranded total RNA-seq kit v.2	Takara	Cat#634412

**Other**		

S220 focused ultrasonicator	Covaris	Cat#500217
microTUBE AFA fiber pre-slit snap-cap 6 × 16 mm	Covaris	Cat#520045
RNA ScreenTape	Agilent	Cat#5067-5576
RNA ScreenTape sample buffer	Agilent	Cat#5067-5577
High sensitivity RNA ScreenTape	Agilent	Cat#5067-5579
High sensitivity RNA ScreenTape sample buffer	Agilent	Cat#5067-5580
High sensitivity D1000 ScreenTape	Agilent	Cat#5067-5584
High sensitivity D1000 sample buffer	Agilent	Cat#5067-5603
Kimble pellet pestle cordless motor	Dwk Life Sciences	Cat# 749540-0000

## Materials and equipment

### Reagent setup

**Table:** 5× IP buffer

Reagent	Final concentration	Amount
Tris–HCl (pH7.5, 1 M)	0.05 *M*	0.25 mL
NaCl (5 M)	0.75 *M*	0.75 mL
Igepal CA-630 (10%)	0.5%	0.25 mL
Nuclease-free water	–	3.75 mL
**Total**	–	**5 mL**

Keep at room temperature (20°C–25°C) until use.

**Table:** 1× IP buffer

Reagent	Final concentration	Amount
5× IP buffer	1×	1 mL
Nuclease-free water	–	4 mL
**Total**	–	**5 mL**

Keep at room temperature (20°C–25°C) until use.

**Table:** Low salt IP buffer

Reagent	Final concentration	Amount
Tris–HCl (pH7.5, 1 M)	0.01 *M*	20 μL
NaCl (5 M)	0.05 *M*	20 μL
Igepal CA-630 (10%)	0.1%	20 μL
Nuclease-free water	–	1940 μL
**Total**	–	**2 mL**

Keep at room temperature (20°C–25°C) until use.

**Table:** High salt IP buffer

Reagent	Final concentration	Amount
Tris–HCl (pH7.5, 1 M)	0.01 *M*	20 μL
NaCl (5 M)	0.5 *M*	200 μL
Igepal CA-630 (10%)	0.1%	20 μL
Nuclease-free water	–	1760 μL
**Total**	–	**2 mL**

Keep at room temperature (20°C–25°C) until use.

### Equipment setup

• Pre-cool centrifuges to 4°C.
• Pre-cool Covaris Sonicator to 4°C.
• When working with RNA, make sure to clean the bench with 70% of ethanol (v/v) and nuclease removal solution, e.g. RNAzap.
• When working with RNA, use filter tip that are sterile.

## Step-by-step method details

### RNA extraction from human postmortem brain tissues

**Timing: 1 h**

The quality of the RNA input significantly affects the identification of high confidence m6A peaks. We compared total RNA^4^ and poly(A)-enriched RNA,^5^ following previously published protocols. Our results showed that total RNA yielded more robust m6A peak profiles with a higher number of identified peaks and stronger enrichment of m6A consensus motif GGAC. (Figure S1: m6A peak profiles generated by total RNA or poly-A RNA). Therefore, this protocol uses total RNA as input following previously published protocol.^4^

This step describes how to prepare total RNA, which serves as the input for immunoprecipitation. We used a commercially available kit to extract total RNA. Ribosomal RNA (rRNA) depletion is not necessary at this stage, as rRNA is effectively removed during the library preparation step.

1. Tissue preparation.
  a. On dry ice, place a weigh boat and 1.5 mL tube to pre-chill.
  b. On the pre-chill weight boat, gently chop frozen tissue using a scalpel.
  c. Measure 40–50 mg of frozen tissue per sample using an analytical scale.
    ***Note:*** Once the frozen tissue is taken out from −80°C, always keep it on dry ice. Keep tissue on dry ice while chopping to minimize degradation and thawing.
  d. Transfer the tissue to a pre-chilled 1.5 mL tube using a spatula and place it immediately on dry ice.
  e. Clean scalpel and spatula with 70% of ethanol (v/v) to avoid contamination between samples.
    **CRITICAL:** Keep frozen tissue on dry ice as much as possible to avoid degradation.
2. Extract RNA from human postmortem brain tissues.
  a. Use RNeasy Lipid Tissue Mini Kit (Qiagen Cat#74804) following the manufacture’s instruction.
    ***Note:*** The manufacturer’s instructions suggest performing tissue disruption and homogenization using either the TissueRuptor II or the TissueLyser. These methods differ in the number of samples that can be processed simultaneously. In our experience, homogenizing each sample with a handheld tissue homogenizer at 2000–3000 rpm for 15 s provided efficient and consistent results.
  b. Following manufacture’s instruction, treat RNA with DNase Digestion.
  c. Following manufacture’s instruction, conduct extra spin before elution step as follow:
    i. Place the RNeasy spin column in a new 2 mL collection tube.
    ii. Centrifuge at full speed for 1 min.
    iii. Move on to elution step.
      ***Note:*** DNase treatment is critical given the presence of m6A on DNA.^12^^,^^13^
      ***Note:*** Make sure to completely remove ethanol by applying extra centrifuge spin, as remaining ethanol could interfere with downstream procedures.
  d. Elute RNA with 40 μL of nuclease-free water.
    **CRITICAL:** Keep RNA on ice to avoid degradation.
    ***Alternatives****:* RNA can be extracted using other method; however, we find this commercial kit perform well for frozen human postmortem brain tissue. It is specifically designed for high-lipid tissues such as brain and consistently yields high amounts of RNA.
  e. Measure RNA concentration and RNA integrity using regular RNA ScreenTape assay with TapeStation (Agilent) following the manufacture’s instruction.
    ***Alternatives:*** RNA samples can also be quantified using other methods such as Bioanalyzer, Qubit, and PicoGreen. We do not recommend using NanoDrop for precise quantification or accurate RNA integrity assessment, as it lacks sensitivity. We highly recommend checking RNA integrity of the sample at this step.
    ***Note:*** When starting from 50 mg of brain tissue, RNA concentration should be in the quantify range of RNA ScreenTape, instead of High Sensitivity RNA ScreenTape.
    **Pause point:** RNA in nuclease-free water can be stored at −80°C until further use. Under these conditions, RNA remains stable for up to one year.

### RNA fragmentation

**Timing: 1 h**

The resolution of m6A peaks largely depend on the size of RNA fragments used for immunoprecipitation, therefore, consistent shearing is critical. This step describes the mechanical fragmentation of total RNA into ∼200-nucleotide fragments using a Covaris Sonicator. The concentration of RNA solution and sonication was optimized to achieve the average fragment length of ∼200-nucleotide.

3. Adjust the RNA concentration for RNA fragmentation.
  a. Add nuclease-free water to adjust the RNA concentration to 200 ng/μL.

**CRITICAL:** Keep RNA on ice to avoid degradation.

4. Fragment RNA using Covaris focused-ultrasonicator.
  a. Set up sonicator (Covaris) and set the water bath temperature to a minimum of 4°C and a maximum of 8°C.
  b. Gently pipette 130 μL of RNA solution into a microTUBE (Covaris).
    ***Note:*** Ensure there are no air bubbles at the bottom of the tube, as they can interfere with sonication efficiency.
    ***Note:*** Fill the tube completely by adding 130 μL of RNA solution, as volume consistency also affects sonication outcomes.
    ***Note:*** Pay attention to when closing the cap to avoid spilling the sample.
  c. Set sonication parameters as follows:

**Table:** 

Target BP (peak)	200
Peak Incident Power (W)	175
Duty Factor	10.0
Cycles per Burst	200
  d. Sonicate samples using the following settings:

**Table:** 

Treatment time (s)	10	20 cycles
Delay (s)	10

    ***Note:*** RNA is prone to degradation. Adding a delay time helps prevent overheating and maintains a low temperature during treatment.
  e. Place the microTUBE in the holder of the sonicator. Ensure the tube is properly aligned and not tilted.
  f. Start sonication cycle.
  g. Once the cycle is complete, carefully remove the microTUBE from the holder and place it on ice.
  h. Using a pipette, transfer RNA solution from the microTUBE to a new 1.5 mL tube.
    ***Note:*** Droplets may remain on the underside of the cap. Be careful not to contaminate the sample with any droplets on the cap and tube wall.
  i. Measure concentration and fragment size using regular RNA ScreenTape assay with TapeStation (Agilent), following the manufacture’s instruction.
    ***Note:*** Confirm that the average fragment size is approximately 200 nt. See Figure 2 for an example. See troubleshootingproblem 1 and 2 if the fragment size is shorter or longer than expected.
  j. Keep a fraction of fragmented RNA on side as an input for input-seq at library preparation step (i.e. the regular RNA-seq library).
    ***Note:*** We recommend keeping at least 50 ng of fragmented RNA when using the library kit described in this protocol. See section library preparation for more details.
    **Pause point:** RNA in nuclease-free water can be stored at −80°C until further use. Under these conditions, RNA remains stable for up to one year.

**Figure 2:**
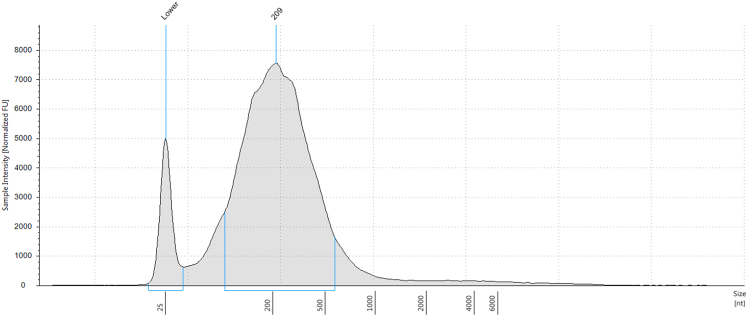
TapeStation trace after RNA fragmentation Representative TapeStation trace showing a peak centered around 200 nt after RNA fragmentation.

### RNA immunoprecipitation

**Timing: 24 h for steps 5 and 6**

**Timing: Timing: 3 h for steps 7 to 11**

The quality of m6A profiles depends greatly on the specificity and affinity of the m6A antibody used. We tested m6A peak characteristic of three commercially available antibodies: the Millipore monoclonal antibody (Cat#MABE1006), the Millipore polyclonal antibody (Cat#ABE572), and the Synaptic Systems polyclonal antibody (Cat#202003) (Figure S2: Comparison of IP efficiency and m6A profiles across different antibodies). This protocol uses the m6A antibody from Synaptic Systems. To ensure high specificity of m6A peak detection, the antibody-to-RNA ratio was optimized for this antibody (Figure S3: Optimization of m6A-IP condition using Synaptic System antibody).

This step describes antibody-bead coupling to prepare for RNA immunoprecipitation.

5. Prepare the magnetic beads.
  a. Place protein A and protein G magnetic beads on an end over and end rotating rack at 24 rpm for 5 min to make sure beads are mixed well before use.
  b. Transfer 30 μL each of protein A and protein G magnetic beads into a new 1.5 mL tube.
    ***Note:*** Prepare a beads-only IP control in parallel to assess non-specific RNA binding. This control includes beads without antibody and helps evaluate background signal caused by non-specific bindings to the beads.
  c. Place the tube on a magnetic stand until all the beads settle and separate completely.
    ***Note:*** The time required for complete separation largely depends on the strength of the magnetic stand used. In our experience, 1 min on a magnetic stand was sufficient when using DynaMag-2 Magnet (Thermo Fisher Scientific Cat#12321D).
  d. Remove and discard the supernatant.
  e. Remove the tube from the magnetic stand.
  f. Wash beads by adding 500 μL of 1× IP buffer (see regent setup) and mix gently by slowly pipetting the liquid along the wall of the tube where the beads adhere.
  g. Repeat the wash once (steps c–f).
6. Make the bead-antibody complex.
  a. After the wash, resuspend the magnetic beads with 500 μL of 1× IP buffer.
  b. Add 1 μg of m6A antibody.
  c. Incubate the tube overnight at 4°C on an end over and end rotating rack at 24 rpm.

***Note:*** Placing a standard rotating rack in a 4°C refrigerator is sufficient. Make sure the tube is securely attached to the rack to prevent it from falling during incubation.

This step describes RNA immunoprecipitation procedure, specifically optimized for Synaptic System antibody to archive high specificity.

7. Prepare bead-antibody-RNA mixture.
  a. Prepare the immunoprecipitation solution as follows. Adjust nuclease-free water to reach a final volume of 500 μL.

**Table:** 

Reagents	Final amount
5× IP buffer (see reagent setup)	100μL
Fragmented RNA	20 μg
RNasin	5 μL
Nuclease-free water	To 500 μL
Total volume	500 μL

***Note:*** Adding RNase inhibitor is important to prevent RNA degradation during immunoprecipitation.

8. Incubate beads with RNA.
  a. Place the tube with beads on a magnetic stand for 1 min and discard supernatant by decanting.
  b. Wash beads by adding 500 μL of 1× IP buffer and mix gently by slowly pipetting the liquid along the wall of the tube.
  c. Discard supernatant by decanting.
  d. Remove tube with bead-antibody complex from the magnet and add the prepared immunoprecipitation solution.
  e. Incubate for 2 h at 4°C on an end over and end rotating rack at 24 rpm.
9. Wash beads.
  a. Place the tube on magnetic stand and discard supernatant by decanting.
  b. Wash beads by adding 500 μL of 1× IP buffer and mix gently by slowly pipetting the liquid along the wall of the tube.
  c. Place tube on an end over and end rotating rack at 24 rpm for 10 min at 4°C.
  d. Place tube on magnetic stand and discard supernatant by decanting.
  e. Repeat wash one time (step b–d).
  f. Was beads by adding 500 μL of low salt IP buffer (see regent setup) and mix gently by slowly pipetting the liquid along the wall of the tube.
  g. Place tube on an end over and end rotating rack at 24 rpm for 10 min at 4°C.
  h. Place tube on magnetic stand and discard supernatant by decanting.
  i. Repeat wash one time (step f–h).
  j. Wash beads by adding 500 μL of high salt IP buffer (see regent setup) and mix gently by slowly pipetting the liquid along the wall of the tube.
  k. Place tube on an end over and end rotating rack at 24 rpm for 10 min at 4°C.
  l. Place tube on magnetic stand and discard supernatant by decanting.
  m. Repeat wash one time (step j–l).

***Note:*** The immunoprecipitation condition, including wash volume and duration, was optimized to increase the specificity of m6A peak enrichment (Figure S3: Optimization of m6A-IP condition using Synaptic System antibody).

10. Elute RNA from antibody-bead-RNA mixture.
  a. Remove the tube from magnetic stand.
  b. Add 50 μL of RLT buffer (QIAGEN) and incubate for 2 min at room temperature.
  c. Place the tube back on the magnetic stand and **transfer** the supernatant to a new 1.5 mL tube. DO NOT DISCARD SUPERNATANT.
  d. Repeat elution (step a–c) with another 50 μL of RLT buffer (QIAGEN) and combine the eluates.

**CRITICAL:** Pay attention not to discard the supernatant. The supernatant contains the eluted IP-RNA.

11. Purify IP yield.
  a. Use RNA clean & concentrator (Zymo Cat#R1015) following the manufacture’s instruction to concentrate and clean IP-RNA.
  b. Elute the purified IP-RNA in 10 μL of nuclease-free water.
    ***Note:*** Keep the elution volume low, as most library preparation kits have a specific amount of input in a relatively small volume, therefore, a high RNA concentration is needed.
  c. Measure the concentration of the IP-RNA using High Sensitivity RNA ScreenTape assay with TapeStation (Agilent) following the manufacture’s instruction.
    ***Note:*** In our experience, the expected IP-RNA yield is around 5–10ng. See troubleshootingproblem 3 and 4 if the IP-RNA is lower or higher than expected.
    **Pause point:** The RNA samples can be stored at −80°C until further use.

### Library preparation

**Timing: 6 h**

This step describes preparation of sequencing libraries from IP-RNA and input sheared RNA.

12. Prepare IP-RNA and comparable amounts of sheared RNA (input control) for library preparation.

***Note:*** When processing multiple samples, ensure the same amount of starting material is used across all samples to maintain consistency and comparability.

***Note:*** The amount of input RNA used for the control should be determined based on the quantity of IP-RNA. In theory, since m6A is highly enriched on mRNA, IP-RNA was enriched for mRNA and depleted for rRNA during immunoprecipitation step. Therefore, a higher amount of fragmented total RNA should be used for the input control to match the amount of mRNA going into the reaction. In our experience, 50ng of fragmented-total-RNA was used as an input control when using 4ng of IP-RNA.

***Note:*** When using the SMARTer Stranded Total RNA-Seq Kit v2 (Takara Cat#634412), the maximum recommended input is 50 ng. Exceeding this amount may exceed the capacity of the rRNA depletion chemistry, leading to an increased proportion of rRNA reads.

**CRITICAL:** It is highly recommended to generate an input control (input-seq) for each sample. This serves as a background reference during m6A peak calling. Proper normalization using input-seq is critical for accurate and reliable peak identification.

13. Prepare sequencing libraries using SMARTer Stranded Total RNA-seq Kit v2 (Takara Cat#634412) following the manufacture’s instruction.

***Note:*** Skip RNA fragmentation step in the protocol as RNA fragmentation was already performed in prior step.

14. Measure the concentration of the library using High Sensitivity D1000 ScreenTape assay with TapeStation (Agilent) following the manufacture’s instruction.

**Figure 3:**
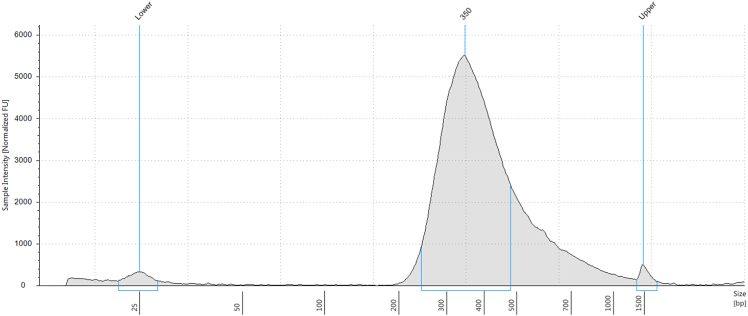
TapeStation trace of m6A-seq library Representative TapeStation trace showing a peak centered around 350 bp for the m6A-seq library.

***Note:*** The number of cycles for library amplification should be selected based on the amount of starting material, ranging from 9–16 cycles. Performing more than 16 cycles is not recommended as it may lead to amplification of background material. In our experience, 16 cycles consistently yield sufficient library concentration for sequencing when starting with 4ng of RNA. See Figure 3 for an example of library trace.

***Note:*** If the library trace is not detectable, refer to troubleshootingproblem 5.

**Pause point:** Libraries can be stored at −80°C for long-term storage.

15. Sequence libraries on an Illumina NovaSeq 6000 platform with 100 bp paired-end mode.

***Alternatives:*** Similar next-generation platform can be used, for example, NovaSeq X Series.

***Note:*** It is recommended to sequence up to 30 million reads per sample. However, studies have suggested there is no direct relationship between peak count and sequencing depth.^6^

## Expected outcomes

Our m6A-seq protocol, optimized for frozen human postmortem brain tissues, is expected to generate high-quality m6A profiles, as demonstrated in our accompanying publication.^1^ The optimized immunoprecipitation conditions should yield RNA-IP with high specificity, enriched for the known m6A consensus motif “GGAC” and localized in the 3′ untranslated region (3′UTR) and coding sequences (CDS), consistent with previous findings.

In addition to experimental optimization, the choice of bioinformatics tools plays a critical role in accurately identifying m6A peaks. Various peak-calling tools and analysis pipelines have been developed. We showed expected m6A profiles using four different peak calling tools—MACS2,^14^ ExomePeak2,^15^ MeRIP,^16^ and TRESS^7^ in the human ventromedial prefrontal cortex (Figure S4: Comparison of peak characteristic by different peak calling methods).

## Limitations

While the antibody-based method remains a common approach for profiling m6A, it has several limitations. As demonstrated, m6A peak profiles are significantly influenced by the choice of antibody and bioinformatics analysis tool (supplemental information). To address these issues, alternative methods such as enzymatic conversion and direct RNA sequencing have been developed.^17^^,^^18^^,^^19^ Unlike the antibody-based method, which identifies m6A peaks at a resolution of 100–200 nucleotides, these alternative methods can detect m6A sites at single-nucleotide resolution. Moreover, antibody-based approaches may fail to capture the full extent of m6A modifications, as direct RNA sequencing has revealed a greater number of m6A sites.^17^ However, further optimization is required to apply these methods to frozen postmortem tissues.

## Troubleshooting

### Problem 1

RNA fragment size shorter than expected. (step 4 in RNA fragmentation).

### Potential solution

• This may be due to inaccurate RNA input concentration.
• This may be due to RNA degradation resulting from heat generated during mechanical shearing, especially if the RNA was not kept at a low temperature throughout the process. Ensure that RNA samples are kept cold, and account for any delay time during shearing to minimize heat exposure and prevent over-fragmentation.

### Problem 2

RNA fragment size longer than expected. (step 4 in RNA fragmentation).

### Potential solution

This may result from inaccurate RNA input concentration or insufficient fragmentation. Add the number of fragmentation cycles (e.g., add 5 additional cycles) and check fragment length again.

### Problem 3

RNA IP yield is lower than expected. (step 11 in RNA immunoprecipitation).

### Potential solution

• RNA may have degraded during immunoprecipitation; always include RNase inhibitors in the IP solution and use RNase-free tubes.
• RNA may not have been fully eluted. Ensure elution is performed at room temperature with adequate incubation time.
• The antibody may not have been added or was improperly handled. Confirm the correct volume was pipetted, and gently mix the antibody before use. Antibody solutions can be viscous and prone to bubble formation—use caution when pipetting small volumes.
• The quality of the input RNA may be low (e.g., RIN < 4). Assess RNA concentration and quality using electrophoretic platforms such as the Bioanalyzer or TapeStation rather than NanoDrop, as these provide more accurate measurements and RNA quality assessment.

### Problem 4

RNA IP yield is higher than expected. (step 11 in RNA immunoprecipitation).

### Potential solution

• This may result from non-specific RNA binding. Use a high-salt IP buffer to reduce non-specific interactions.
• To evaluate binding specificity, measure the signal-to-noise ratio of the IP yield based on previously published protocols before proceeding to sequencing.^4^ (see Figures S2 and S3).

### Problem 5

No detectable libraries in the final library trace. (step 14 in library preparation).

### Potential solution

• This may be due to insufficient RNA input during library preparation. Ensure RNA concentration is accurately measured and adjust input amounts as recommended by the library prep kit.

## Resource availability

### Lead contact

Further information and requests for resources and reagents should be made directly to, and will be fulfilled by, Gustavo Turecki (gustavo.turecki@mcgill.ca).

### Technical contact

Technical questions on executing this protocol should be directed to and will be answered by the technical contact, Corina Nagy (corina.nagy@mcgill.ca).

### Materials availability

This study did not generate new unique reagents.

### Data and code availability

The raw sequencing data generated using this protocol have been deposited at GEO and are publicly available. The accession number can be found in the key resources table. This study did not generate original code. Any additional information required to reanalyze the data reported in this paper is available from the lead contact upon request.

## Acknowledgments

G.T. holds a Canada Research Chair (Tier 1) and is supported by grants from the Canadian Institutes of Health Research (CIHR; FDN148374, ENP161427 [ERA-NET ERA PerMed], PJT183903, and PJT189993) and National Institutes of Health (NIH; R56MH131818-01). C.N. holds an FRQS research scholar junior 1. We acknowledge the experts at Douglas-Bell Canada Brain Bank. This Project has been made possible with the financial support of Health Canada, through the Canada Brain Research Fund, an innovative partnership between the Government of Canada (through Health Canada) and Brain Canada, and in part by funding from the Canada First Research Excellence Fund, awarded to McGill University for the Healthy Brains for Healthy Lives initiative, and from the Fonds de recherche du Québec - Santé (FRQS) through the Quebec Network on Suicide, Mood Disorders and Related Disorders. This research was enabled in part by support provided by Calcul Québec (https://www.calculquebec.ca/en/) and the Digital Research Alliance of Canada (alliancecan.ca).

## Author contributions

H.M. led data curation, data analysis, data investigation, and writing. N.M. contributed to sample procurement. C.N. and G.T. supervised the study design, data investigation, and writing. G.T. obtained funding.

## Declaration of interests

The authors declare no competing interests.

## Footnotes

Supplemental information can be found online at https://doi.org/10.1016/j.xpro.2025.104149.

## References

[1] Mitsuhashi H., Lin R., Chawla A., Mechawar N., Nagy C., Turecki G. (2024). Altered m6A RNA methylation profiles in depression implicate the dysregulation of discrete cellular functions in males and females. iScience.

[2] Dominissini D., Moshitch-Moshkovitz S., Schwartz S., Salmon-Divon M., Ungar L., Osenberg S., Cesarkas K., Jacob-Hirsch J., Amariglio N., Kupiec M. (2012). Topology of the human and mouse m6A RNA methylomes revealed by m6A-seq. Nature.

[3] Meyer K.D., Saletore Y., Zumbo P., Elemento O., Mason C.E., Jaffrey S.R. (2012). Comprehensive analysis of mRNA methylation reveals enrichment in 3' UTRs and near stop codons. Cell.

[4] Zeng Y., Wang S., Gao S., Soares F., Ahmed M., Guo H., Wang M., Hua J.T., Guan J., Moran M.F. (2018). Refined RIP-seq protocol for epitranscriptome analysis with low input materials. PLoS Biol..

[5] Merkurjev D., Hong W.T., Iida K., Oomoto I., Goldie B.J., Yamaguti H., Ohara T., Kawaguchi S.Y., Hirano T., Martin K.C. (2018). Synaptic N^6^-methyladenosine (m^6^A) epitranscriptome reveals functional partitioning of localized transcripts. Nat. Neurosci..

[6] McIntyre A.B.R., Gokhale N.S., Cerchietti L., Jaffrey S.R., Horner S.M., Mason C.E. (2020). Limits in the detection of m6A changes using MeRIP/m6A-seq. Sci. Rep..

[7] Guo Z., Shafik A.M., Jin P., Wu H. (2022). Differential RNA methylation analysis for MeRIP-seq data under general experimental design. Bioinformatics.

[8] Zhang Z., Zhan Q., Eckert M., Zhu A., Chryplewicz A., De Jesus D.F., Ren D., Kulkarni R.N., Lengyel E., He C., Chen M. (2019). RADAR: Differential analysis of MeRIP-seq data with a random effect model. Genome Biol..

[9] Sidova M., Tomankova S., Abaffy P., Kubista M., Sindelka R. (2015/09). Effects of post-mortem and physical degradation on RNA integrity and quality. Biomol. Detect. Quantif..

[10] Nagy C., Maheu M., Lopez J.P., Vaillancourt K., Cruceanu C., Gross J.A., Arnovitz M., Mechawar N., Turecki G. (2015). Effects of Postmortem Interval on Biomolecule Integrity in the Brain. J. Neuropathol. Exp. Neurol..

[11] Schoenberg D.R. (2011). Mechanisms of endonuclease-mediated mRNA decay. Wiley Interdiscip. Rev. RNA.

[12] Wu T.P., Wang T., Seetin M.G., Lai Y., Zhu S., Lin K., Liu Y., Byrum S.D., Mackintosh S.G., Zhong M. (2016). DNA methylation on N(6)-adenine in mammalian embryonic stem cells. Nature.

[13] Conti B.A., Novikov L., Tong D., Xiang Q., Vigil S., McLellan T.J., Nguyen C., De La Cruz N., Veettil R.T., Pradhan P. (2025). N6-methyladenosine in DNA promotes genome stability. eLife.

[14] Zhang Y., Liu T., Meyer C.A., Eeckhoute J., Johnson D.S., Bernstein B.E., Nusbaum C., Myers R.M., Brown M., Li W., Liu X.S. (2008). Model-based analysis of ChIP-Seq (MACS). Genome Biol..

[15] Meng J., Lu Z., Liu H., Zhang L., Zhang S., Chen Y., Rao M.K., Huang Y. (2014). A protocol for RNA methylation differential analysis with MeRIP-Seq data and exomePeak R/Bioconductor package. Methods.

[16] Liu S., Zhu A., He C., Chen M. (2020). REPIC: A database for exploring the N 6-methyladenosine methylome. Genome Biol..

[17] Hendra C., Pratanwanich P.N., Wan Y.K., Goh W.S.S., Thiery A., Göke J. (2022). Detection of m6A from direct RNA sequencing using a multiple instance learning framework. Nat. Methods.

[18] Ge R., Ye C., Peng Y., Dai Q., Zhao Y., Liu S., Wang P., Hu L., He C. (2023). m(6)A-SAC-seq for quantitative whole transcriptome m(6)A profiling. Nat. Protoc..

[19] Meyer K.D. (2019). DART-seq: an antibody-free method for global m6A detection. Nat. Methods.

