# Supplementary material for: Protocol for antibody-based m6A sequencing of human postmortem brain tissues
Source: STAR Protoc. 2025 Oct 17;6(4):104149. doi: 10.1016/j.xpro.2025.104149 (PMC12556178; doi:10.1016/j.xpro.2025.104149)
Supplement: Document S1. Figures S1–S4 [file mmc1.pdf]

Figure S1

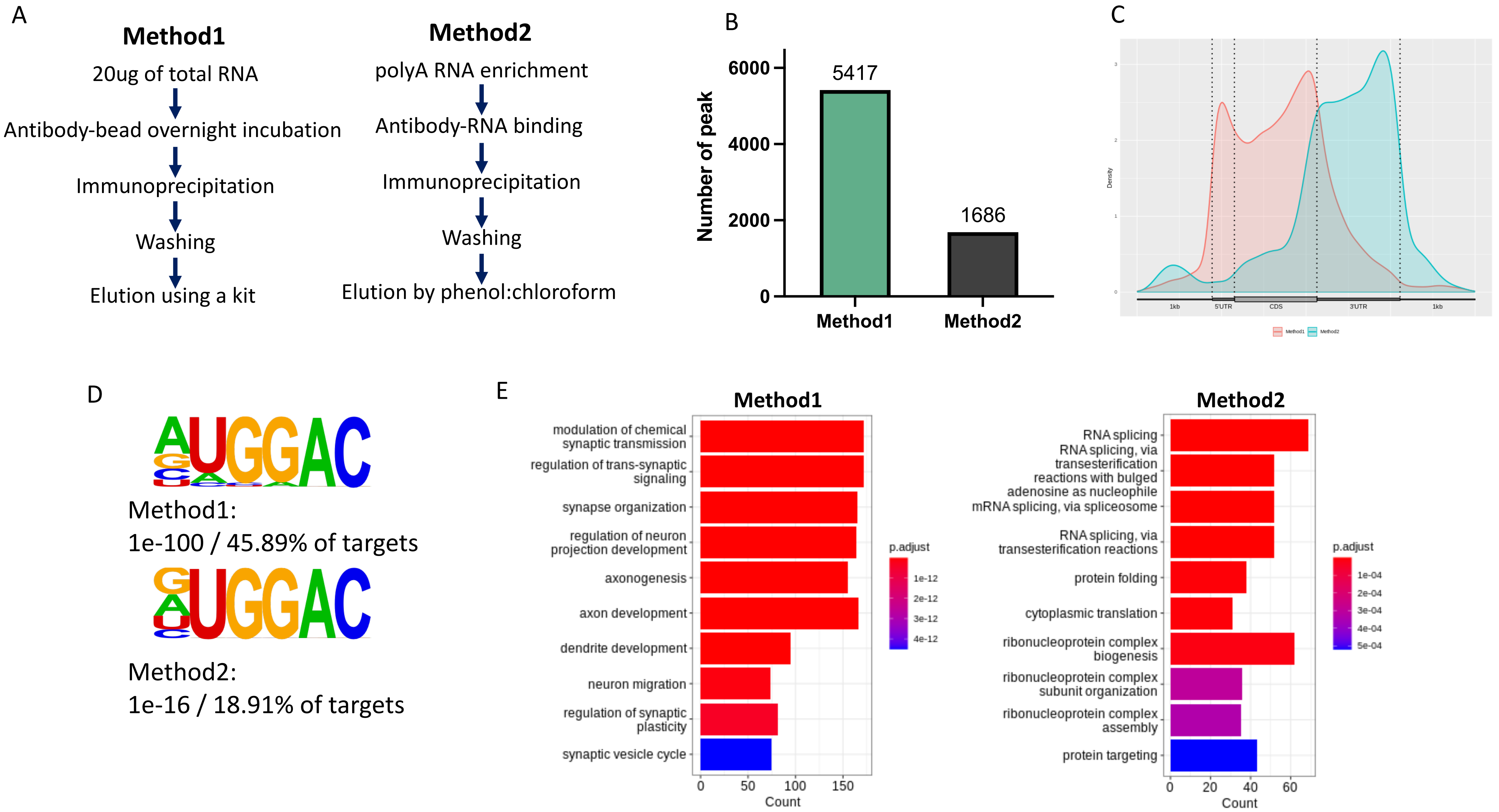
A
Method1
20ug of total RNA
Antibody-bead overnight incubation
Immunoprecipitation
Washing
Elution using a kit
Method2
polyA RNA enrichment
Antibody-RNA binding
Immunoprecipitation
Washing
Elution by phenol:chloroform
B
Number of peak
6000
4000
2000
0
5417
1686
Method1
Method2
C
Density
3
2
1
0
1kb
5'UTR
CDS
3'UTR
1kb
Method1
Method2
D
AUGGAC
Method1:
1e-100 / 45.89% of targets
UGGAC
Method2:
1e-16 / 18.91% of targets
E
Method1
modulation of chemical synaptic transmission
regulation of trans-synaptic signaling
synapse organization
regulation of neuron projection development
axonogenesis
axon development
dendrite development
neuron migration
regulation of synaptic plasticity
synaptic vesicle cycle
0
50
100
150
Count
p.adjust
1e-12
2e-12
3e-12
4e-12
Method2
RNA splicing
RNA splicing, via transesterification reactions with bulged adenosine as nucleophile
mRNA splicing, via spliceosome
RNA splicing, via transesterification reactions
protein folding
cytoplasmic translation
ribonucleoprotein complex biogenesis
ribonucleoprotein complex subunit organization
ribonucleoprotein complex assembly
protein targeting
0
20
40
60
Count
p.adjust
1e-04
2e-04
3e-04
4e-04
5e-04

Figure S2

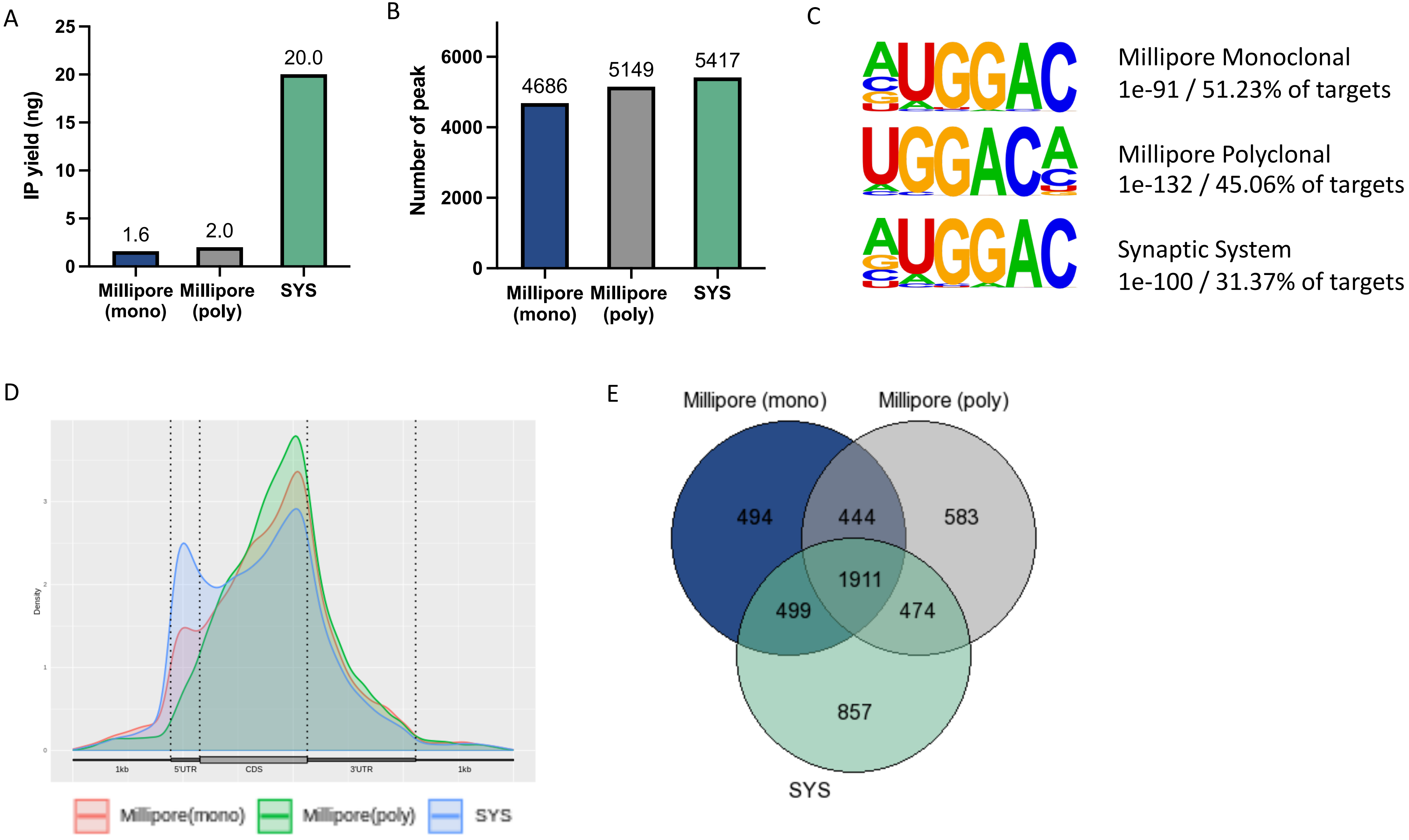
A
IP yield (ng)
25
20
15
10
5
0
1.6
2.0
20.0
Millipore (mono)
Millipore (poly)
SYS
B
Number of peak
6000
4000
2000
0
4686
5149
5417
Millipore (mono)
Millipore (poly)
SYS
C
AUGGAC
Millipore Monoclonal
1e-91 / 51.23% of targets
UGGACA
Millipore Polyclonal
1e-132 / 45.06% of targets
AUGGAC
Synaptic System
1e-100 / 31.37% of targets
D
Density
1kb
5'UTR
CDS
3'UTR
1kb
Millipore(mono)
Millipore(poly)
SYS
E
Millipore (mono)
Millipore (poly)
494
444
583
1911
499
474
857
SYS

Figure S3

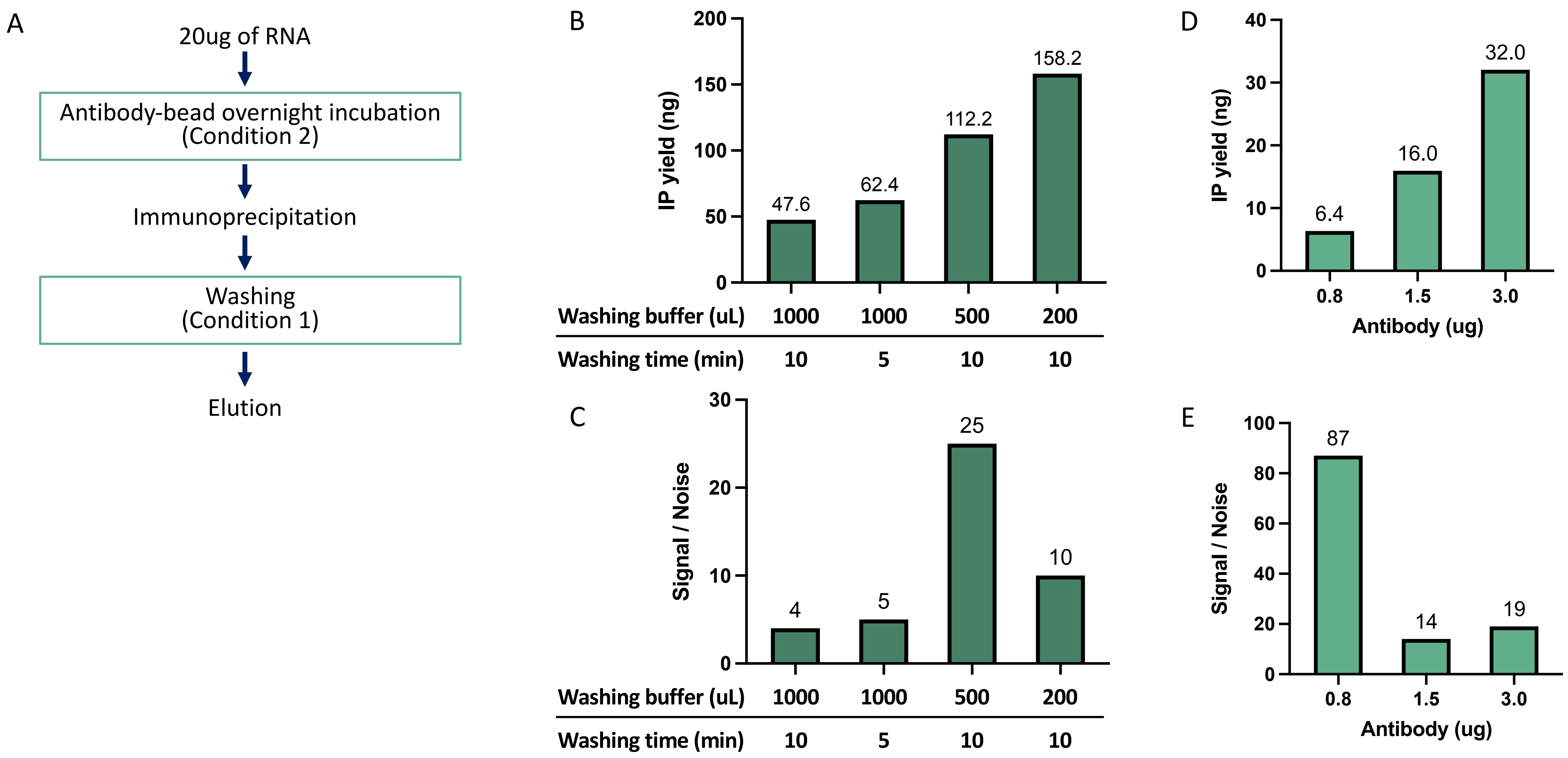
A
20ug of RNA
Antibody-bead overnight incubation
(Condition 2)
Immunoprecipitation
Washing
(Condition 1)
Elution
B
IP yield (ng)
200
150
100
50
0
47.6
62.4
112.2
158.2
Washing buffer (uL) 1000 1000 500 200
Washing time (min) 10 5 10 10
C
Signal / Noise
30
20
10
0
4
5
25
10
Washing buffer (uL) 1000 1000 500 200
Washing time (min) 10 5 10 10
D
IP yield (ng)
40
30
20
10
0
6.4
16.0
32.0
0.8 1.5 3.0
Antibody (ug)
E
Signal / Noise
100
80
60
40
20
0
87
14
19
0.8 1.5 3.0
Antibody (ug)

Figure S4

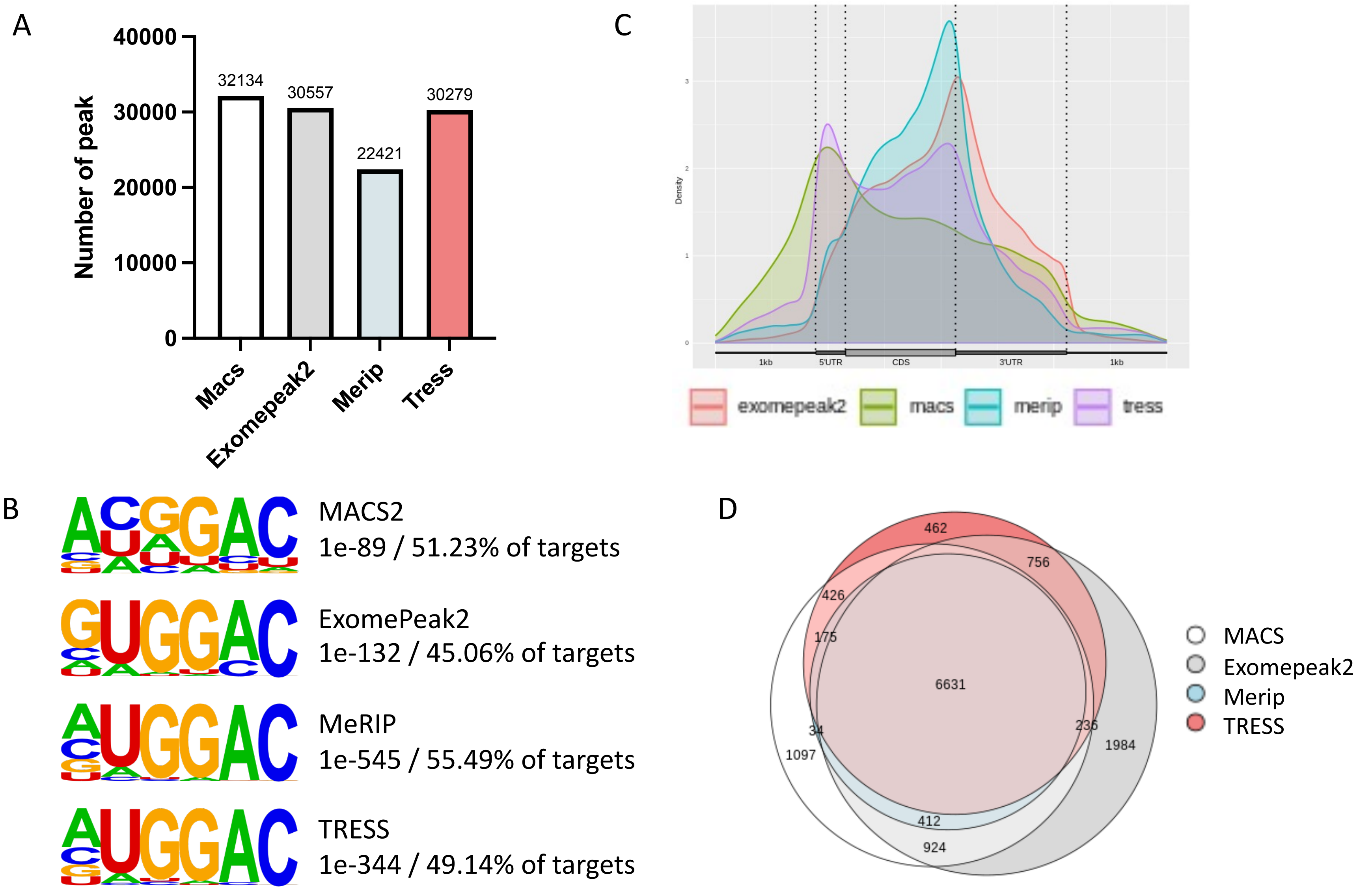

A
Number of peak
40000
30000
20000
10000
0
32134
30557
22421
30279
Macs
Exomepeak2
Merip
Tress
B
MACS2
1e-89 / 51.23% of targets
ExomePeak2
1e-132 / 45.06% of targets
MeRIP
1e-545 / 55.49% of targets
TRESS
1e-344 / 49.14% of targets
C
Density
1kb
5'UTR
CDS
3'UTR
1kb
exomepeak2
macs
merip
tress
D
462
756
426
175
6631
34
236
1097
1984
412
924
MACS
Exomepeak2
Merip
TRESS

Figure S1: m6A peak profiles generated by total RNA or poly-A RNA, related step Extract RNA from human postmortem brain tissues.
(A) Experimental flow of immunoprecipitation tested using total RNA (Method1) or poly-A RNA (Method2) as an input (n=1).
(B) Bar plot showing the number of called m6A peaks identified using TRESS.
(C) Distribution of m6A peaks across transcript regions, with Method 1 showing enrichment in the CDS, while Method 2 shows enrichment in the 3′ UTR.
(D) Motif enrichment analysis showing stronger enrichment of the "GGAC" motif in Method 1.
(E) Gene ontology in biological process analysis showing greater enrichment of synaptic function–related genes in Method 1 (left), whereas Method 2 (right) shows enrichment in genes related to general RNA function.

Figure S2: Comparison of IP efficiency and m6A profiles across different antibodies, related to step Antibody-Bead coupling.
(A) Bar plot showing the IP yield (ng) obtained using different antibodies (n = 1).
(B) Using the IP yield from (A), m6A-seq was performed (n = 1). Bar plot displaying the number of peaks identified by TRESS for each antibody.
(C) The top motif identified across datasets, with its associated p-value and the percentage of target sites, showing significant enrichment for the GGAC motif. The Synaptic Systems (SYS) antibody showed the lowest percentage of motif-enriched peaks. Overall, the SYS antibody may exhibit lower specificity compared to Millipore antibodies. However, the polyclonal Millipore antibody, which showed the highest specificity, has been discontinued. Since the SYS antibody remains one of the most widely used in the field, this protocol proceeded using the SYS antibody.
(D) Distribution of m6A peaks across transcript regions, indicating enrichment in the 3′ UTR. The SYS antibody showed higher enrichment in the 5′ UTR compared to Millipore antibodies.
(E) Venn diagram showing the overlap of m6A-tagged genes detected across different antibodies.

Figure S3: Optimization of m6A-IP condition using Synaptic System antibody, related to step Antibody-Bead coupling.
(A) Immunoprecipitation conditions using the SYS antibody were tested by varying the washing stringency (Condition 1) and the amount of antibody used in the reaction (Condition 2) (n=1).
(B, C) Bar plots showing the IP yield (B) and signal-to-noise ratio (C) under different washing conditions (Condition 1). The IP yield increased with less stringent washing. Signal-to-noise ratio was calculated using SETD7 serving as a positive control and GAPDH as a negative control, according to the previous publication [4].
(D, E) Bar plots showing the IP yield (D) and signal-to-noise ratio (E) under varying antibody amounts (Condition 2). The IP yield correlated with the amount of antibody used.

Figure S4: Comparison of peak characteristic by different peak calling methods, related to Expected outcomes.
(A) Bar plots showing the number of m6A peaks identified by each peak-calling tool.
(B) Distribution of m6A peaks identified by each tool across transcript regions.
(C) Enrichment of the “GGAC” motif detected by each peak-calling tool.
(D) Number of m6A-tagged genes identified by each tool.
